# Parkinson's Disease Remote Patient Monitoring During the COVID-19 Lockdown

**DOI:** 10.3389/fneur.2020.567413

**Published:** 2020-10-07

**Authors:** Francesco Motolese, Alessandro Magliozzi, Fiorella Puttini, Mariagrazia Rossi, Fioravante Capone, Keren Karlinski, Alit Stark-Inbar, Ziv Yekutieli, Vincenzo Di Lazzaro, Massimo Marano

**Affiliations:** ^1^Neurology, Neurophysiology and Neurobiology Unit, Department of Medicine, University Campus Bio-Medico of Rome, Rome, Italy; ^2^Montfort Brain Monitor, Binyamina, Israel

**Keywords:** Parkinson's disease (PD), remote patient monitoring (RPM), telemedicine, smartphone, COVID

## Abstract

**Objective:** To evaluate the feasibility of a smartphone remote patient monitoring approach in a real-life Parkinson's disease (PD) cohort during the Italian COVID-19 lockdown.

**Methods:** Fifty-four non-demented PD patients who were supposed to attend the outpatient March clinic were recruited for a prospective study. All patients had a known UPDRS-III and a modified Hoehn and Yahr (H&Y) score and were provided with a smartphone application capable of providing indicators of gait, tapping, tremor, memory and executive functions. Different questionnaires exploring non-motor symptoms and quality of life were administered through phone-calls. Patients were asked to run the app at least twice per week (i.e., full compliance). Subjects were phone-checked weekly throughout a 3-week period for compliance and final satisfaction questionnaires.

**Results:** Forty-five patients (83.3%) ran the app at least once; Twenty-nine (53.7%) subjects were half-compliant, while 16 (29.6%) were fully compliant. Adherence was hindered by technical issues or digital illiteracy (38.7%), demotivation (24%) and health-related issues (7.4%). Ten patients (18.5%) underwent PD therapy changes. The main factors related to lack of compliance included loss of interest, sadness, anxiety, the absence of a caregiver, the presence of falls and higher H&Y. Gait, tapping, tremor and cognitive application outcomes were correlated to disease duration, UPDRS-III and H&Y.

**Discussion:** The majority of patients were compliant and satisfied by the provided monitoring program. Some of the application outcomes were statistically correlated to clinical parameters, but further validation is required. Our pilot study suggested that the available technologies could be readily implemented even with the current population's technical and intellectual resources.

## Introduction

On March 9th, 2020 the Italian government imposed a national lockdown, due to Coronavirus disease 2019 (COVID-19) outbreak. Such restriction also aimed to protect fragile people with chronic diseases, a population that is particularly at risk of SARS-CoV-2 complications. However, these patients often needed a tight follow-up and therapies to be tailored from time-to-time. In the last few years, mobile technologies have been extensively explored in patient management. However, this has not changed current clinical practices (1). Herein, we present a prospective study in which we explored the feasibility of remote patient monitoring (RPM) in a real-life cohort of Parkinson's disease (PD) patients. This was performed through a smartphone application designed for monitoring motor and cognitive performances of patients affected by neurological disorders.

## Patients and Methods

Non-demented PD patients, who were supposed to attend the outpatient clinic in March 2020 for follow-up visits and owned a smartphone, were recruited. All subjects who were enrolled in this observational study received a first phone-call to collect information about their sociodemographic data, their baseline PD motor and non-motor status and quality of life. Accordingly, the following questionnaires were adopted: the Non-Motor Symptoms Questionnaire (NMSQ), the Unified Parkinson's Disease Rating Scale for mentation behavior and mood, activities of daily living and complications of therapy (UPDRS I, II, and IV respectively), the Geriatric Depression Scale short form (GDSsf) and the Parkinson's Disease Questionnaire-8 (PDQ8) (2).

All questionnaires were collected by an experienced clinician (MM) and a trained rater (FP) and the phone-calls were delivered directly to the patient, with or without the involvement of the caregiver.

In the same phone-call, all patients were provided with the instructions to download, run and use the EncephaLog Home™ smartphone application. We provided all the necessary instructions for the use of the app both in written form (i.e., through supportive emails) and a video instruction embedded in the app itself (i.e., only for the TUG test). However, patients were allowed to receive caregivers' help whenever needed.

The app included a starting question with a self-evaluation of the global “Parkinson Status” (0–5), followed by a sequence of cognitive tests exploring reaction time, interference and memory and 10 consecutive tasks exploring motor functions (postural and rest tremor for both arms, timed tapping test for both hands, balance assessment in neutral stance and feet together and two 3-meters Time-Up-and-Go or TUG test). It took ~15 to 20 min to carry out all the tasks included in the app.

Patients were asked to use the “app” at least twice a week for a 3-week observation period, but they were allowed to use it (as unsolicited) as needed to let the neurologist track their status. Subjects were phone-checked weekly throughout a 3-week period for compliance, upcoming issues and for an evaluation questionnaire at the end of the observation period. The latter was sent to patients by email and mailed back to the physician via e-mail or regular mail. Further details on the final evaluation questionnaires are reported in Supplementary Materials.

Data of the last available in-person motor status (i.e., performed in the hospital) was retrospectively collected from medical records. This included the UPDRS-III total score and the modified Hoehn and Yahr scale (H&Y) (3). Both were rated by a single trained physician (MM).

EncephaLog Home™ is a smartphone application, supported both by iOS and Android operating systems, designed by Montfort Brain Monitor LTD (https://www.mon4t.com) - a company providing smartphone-based neurological tests. The English native app was translated in Italian by Montfort (ZY, KK, AS) with medical scientific counseling provided by the Neurology, Neurobiology and Neurophysiology unit of Campus Bio-Medico of Rome University (FM, FP, AM, MM). Further descriptions of the app, its validation stage and details of tests are reported in the Supplementary Materials.

All individuals provided informed consent in regards to their participation to the study. The research was performed according to the Declaration of Helsinki and approved by the ethic committee of Campus Bio-Medico of Rome University.

Anonymized app data was prospectively collected and sent from the smartphone in a secured manner (using HTTPS), using Azure for storing and processing the raw data. The latter was accessible along the study but was analyzed only at the end. When the research was conducted, the app data was not meant to be used as an aid to support any kind of intervention (e.g., medication changes).

Descriptive statistics are reported as frequencies (%) or median (quartiles, QI-QIII). Inferential statistics were carried out by the Wilcoxon/Kruskal-Wallis or the Chi-squared test according to data and distributions. The association between variables was investigated by the Spearman test and described as correlation coefficient (*p*-value). A *p* < 0.05 was adopted as a cut-off to determine statistical significance. Statistics were performed by the JMP-14 software (SAS institute Inc.).

Anonymized data can be made available to qualified investigators.

## Results

Fifty-four consecutive PD patients were enrolled, see Table 1 for socio-demographic and disease feature baseline. Eight patients preferred not to disclose their economic status by phone-calls. Most of them had a caregiver involved in the PD care (46, 85%). Caregivers showed a younger median age (48, 37.5–69.2; *p* < 0.001) and a trend of having a higher formal education level (*p* = 0.066) than patients.

**Table 1:** Population's socio-demographic and disease feature baseline data.

Age (years)	66.5 (59.7–72.2)
Sex (F), *n* (%)	18 (33)
**Education**	
Bachelor's degree, *n* (%)	15 (27.7)
High school, *n* (%)	26 (48.1)
Upper secondary school or lower, *n* (%)	13 (22)
**Annual family income**	
> 55.000 €, *n* (%)	7 (13)
28–55.000 €, *n* (%)	15 (27.5)
<28.000 €, *n* (%)	24 (44)
Not provided	8 (15)
**Comorbidities**	
3 or more, *n* (%)	17 (31.5)
1 or 2, *n* (%)	21 (38.8)
None, *n* (%)	16 (29.6)
**Presence of caregiver**	
Close relative (Spouse or son)	43 (79.6)
Other relative or close friends	3 (5.5)
None	8 (14.8)
**Caregiver education**	
Bachelor's degree, *n* (%)	20 (43.4)
High school, *n* (%)	12 (66)
Upper secondary school or lower, *n* (%)	14 (30)
Disease duration (years)	6.5 (4–11)
Modified Hoehn & Yahr scale	2.5 (2–3)
Patients on Levodopa, *n* (%)	39 (80%)
LEDD (mgs)	547.5 (366.25–1,061.25)
**Patients on advanced therapies**	
STN DBS, *n* (%)	5 (9.2)
LCIG, *n* (%)	11 (20.3)
UPDRS-III total score	22 (14–32)
UPDRS-I total score	1 (0–2)
UPDRS-II total score	11 (7–16)
UPDRS-IV A & B total score	2 (0–3)
NMSQ total score	9 (5.75–13)
GDSsf total score	3 (1–7)
PDQ8 score (%)	18.8 (9.4–31.3)

*Data is reported as median (quartiles, QI-QIII) or frequencies (%). LEDD, Levodopa Equivalency Daily Dose; STN DBS, subthalamic nucleus deep brain stimulation; LCIG, levodopa-carbidopa intestinal gel; UPDRS, Unified Parkinson's Disease Rating Scale; NMSQ, Non-Motor Symptoms Questionnaire; GDSsf, Geriatric Depression Scale short form; PDQ8, Parkinson's Disease Questionnaire 8*.

Retrospective UPDRS-III total and H&Y data was traced back no farther than 6 months.

Forty-five (83.3%) patients used the app at least once throughout the entire follow-up period of 3 weeks, with a total number of 313 accesses to tests.

**Figure 1:**
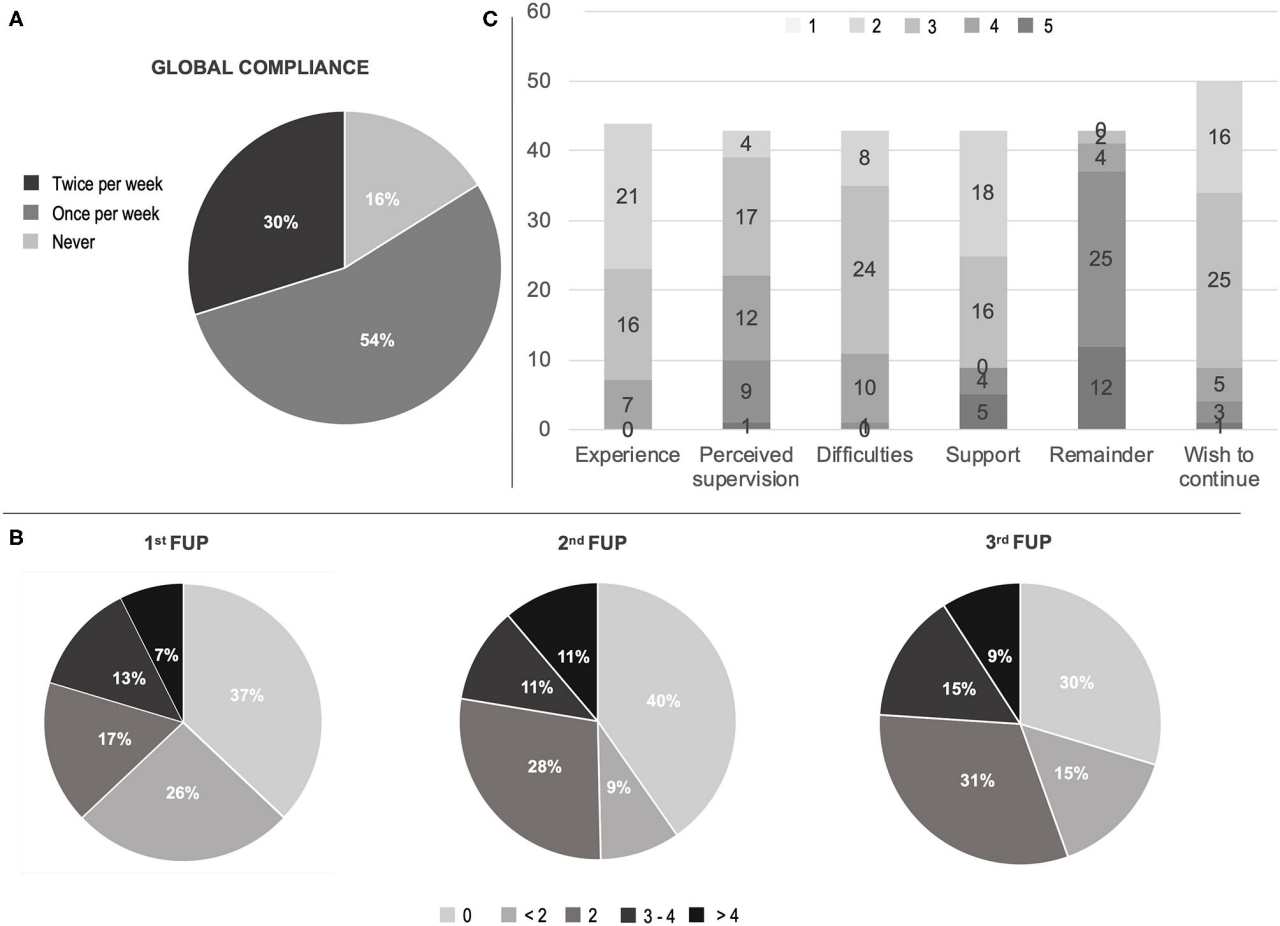
Compliance and satisfaction questionnaire results. **(A)** Global compliance; **(B)** First to third week check-point for compliance (times of app utilization for each week); **(C)** Visual analog scale (VAS) on satisfaction questionnaire experience, perceived medical control, difficulties in using the app, need of support in using the app, burden by app remaining, wish to continue the remote patient monitoring. Scores in C ranged in ascending order from one (light gray) to five (dark gray) according to patient satisfaction. FUP, follow-up call.

In reference to compliance, 29 (53.7%) subjects used the app at least once per week for the 3-week observation period, while 16 (29.6%) were fully compliant (i.e., ran the test at least twice each week for the 3-week observation time) (Figure 1). Compliance was hindered by technical issues or digital illiteracy (21, 38.8%), demotivation or non-specific compliance loss (13, 24%) and health-related issues (4, 7.4% with 1 COVID-19 case). In only two cases (3.7%) technical difficulties—i.e., old-generation smartphones—and digital illiteracy impeded the use of the app; all other issues were solved through phone support. Ten patients (18.5%) underwent PD treatment changes, upon request due to clinical reasons. All performed therapeutic interventions were routine modifications of ongoing medications. None was driven by the app outcomes, due to the observational nature of the study at this stage.

Socio-demographic factors did not relate to the compliance, with the exception of caregiver presence. The latter was tendentially associated to a higher rate of full compliance (*p* = 0.051), as well as to a better adherence throughout the program (*p* = 0.029 at the 3rd phone-check)

PD medications, the presence of advanced therapies (i.e., deep brain stimulation or infusional therapies), UPDRS-I and II total scores did not relate to compliance. However, patients undergoing therapy modifications were most likely fully compliant (*p* = 0.005), as well as patients with motor fluctuations (UPDRS-IV, sudden or unpredictable offs, *p* = 0.038). On the other hand, patients with loss of interest on NMSQ were, in the majority of cases, not fully compliant (*p* = 0.020, respectively). Similarly, NMSQ loss of interest (*p* = 0.024), sadness (*p* = 0.048) and anxiety (*p* = 0.019) were related to low adherence on the 1st-check, while lack of motivation (UPDRS-I) related to a later loss of compliance on the 3rd (*p* = 0.007) phone-check. The presence of falls (UPDRS-II; *p* = 0.019) and of a higher H&Y (*p* = 0.008) were related to a lower compliance rate on the 3rd phone-check (Table 2).

**Table 2:** Motor and non-motor related issues and compliance to the remote patient monitoring prescription.

**Full compliance**	**No**		**Yes**			**p-value**
**Medication changes**						
No	33 (64.7)		8 (15.7)			0.005
Yes	2 (3.9)		8 (15.7)			
**UPDRS-IV, sudden or unpredictable offs**						
No	32 (62.7)		11 (21.6)			0.038
Yes	3 (5.9)		5 (9.8)			
**NMSQ, Loss of interest**						
No	23 (45)		15 (29)			0.020
Yes	12 (25.5)		1 (2)			
**1st compliance check (*****n*** **of app usage)**	**0**	**1**	**2**	**3-4**	**>4**	***p*****-value**
**NMSQ, Loss of interest**						
No	14 (27.5)	7 (13.7)	6 (11.7)	7 (13.7)	4 (7.8)	0.024
Yes	3 (5.8)	7 (13.7)	3 (5.8)	0	0	
**NMSQ, Sadness**						
No	12 (23.5)	6 (11.8)	3 (5.8)	5 (9.8)	4 (7.8)	0.048
Yes	5 (9.8)	8 (15.7)	6 (11.7)	2 (3.9)	0	
**NMSQ, Anxiety**						
No	10 (19.6)	9 (17.6)	2 (3.9)	6 (11.7)	4 (7.8)	0.193
Yes	7 (13.7)	5 (9.8)	7 (13.7)	1 (1.9)	0	
**3rd compliance check (*****n*** **of app usage)**	**0**	**1**	**2**	**3–4**	**>4**	***p*****-value**
**UPDRS-I, lack of motivation**						
Normal	12 (23.5)	2 (3.9)	15 (29.4)	7 (13.7)	4 (7.8)	0.007
Less assertive	1 (1.96)	1 (1.96)	2 (3.9)	0	1 (1.96)	
Loss of initiative in elective activities.	0		0	1 (1.96)	0	
Loss of initiative in day to day activities.	0	1 (1.96)	0	0	0	
**UPDRS-II, Falling**						
None	12 (23.5)	4 (7.8)	14 (27.5)	3 (5.8)	4 (7.8)	0.019
Rare falling	1 (1.9)	4 (7.8)	0	3 (5.8)	1 (1.9)	
Less than once per day	0	0	3 (5.8)	1 (1.9)	0	
Once daily	0	0	0	1 (1.9)	0	
**Modified Hoehn & Yahr scale**						
1–2	2 (3.9)	4 (7.8)	8 (15.7)	0	5 (9.8)	0.008
2.5	8 (15.7)	1 (1.9)	4 (7.8)	5 (9.8)	0	
3–4	3 (5.8)	3 (5.8)	5 (9.8)	3 (5.8)	0	

*UPDRS, Unified Parkinson's Disease Rating Scale; NMSQ, Non-Motor Symptoms Questionnaire; Data is presented as numbers, total frequencies (%). Missing data: there are 3 missing evaluation per compliance check*.

The analysis of data coming from the final evaluation questionnaire showed that 37 (84%) subjects evaluated their experience as “satisfying” (16, 36.4%) or “very satisfying” (21, 48%) and 21 cases (48%) perceived themselves as “safer” (17, 39.5%) or “much safe” (4, 9.3%) thanks to the RPM. However, 17 (37.2%) required “occasional” support and 9 (21%) “frequent to regular” support by the caregiver. Similarly, a minority (11, 26%) perceived the app as difficult (Figure 1, Supplementary Table).

An overview of the data collected from the app and the correlation between the app outcomes and disease duration, UPDRS-III total and H&Y are presented in Supplementary Tables.

## Discussion

This study reported the feasibility of a smartphone-based RPM in a real-life cohort of non-demented PD patients, who were unable to attend the regular follow-up visits due to COVID-19 pandemic.

The “full compliance” (i.e., running the app at least twice per week, for the 3-week period) was an ambitious target to achieve. However, It was encountered in ~30% and was significantly associated to therapeutic changes and to the presence of motor fluctuations. Although the present RPM study was not designed to perform any medical intervention, such result might suggest that a “patient-demanded” remote monitoring is better suited for “active” follow-ups more than for “passive” at-home monitoring. Nevertheless, more than a half of subjects (~55%) spontaneously performed the full ~20-min assessment weekly, providing useful data to track their motor and non-motor performances. This result should not be underestimated in light of future potential studies on disease phenotypes and progression tracking.

Our data shares similarities with previous studies. For instance, Arora et al. obtained a 68% adherence by a sample of 10 mild-to-moderate, well-educated, PD patients (vs. 10 controls). All of them received a smartphone with a 5-min/5-task application, to be performed 4 times a day for a month (4). A ~65% adherence rate was reported also by Horin et al. on a sample of 20 mild-to-moderate PD patients, who were asked to perform a 30-min daily monitoring of three domains on their own smartphone for 90 days (5).

In our study, the majority of patients were compliant and satisfied (Figure 1). However, technical difficulties had an incidence close to ~40%, which is in line with the Italian data on population's problem solving skills in a technological environment (6). Hence, it is reasonable to affirm that, with an adequate in-person training, the program adherence could have been even higher.

Moreover, due to the real-life prospective design, our sample did not exclude patients with a severe involvement (5% had a H&Y of 4) or a lower instruction (22%), being representative of the entire non-demented PD population even on a socio-demographical point-of-view. Additionally, both the contingency of COVID-19 national lockdown and patients' emotional profile might have influenced the adherence. For instance, some non-motor aspects were associated to compliance at the beginning of the RPM (i.e., loss of interest, sadness and anxiety), while others had a prominent role in the full-term program adherence (i.e., lack of motivation, a more severe motor profile). The presence of an educated caregiver is considered essential, nowadays, in the care of PD patients (7). Their role in PD-related device management has been already acknowledged. For instance, the presence of a caregiver in advanced PD patients on device-aided therapies was associated to a better therapeutic outcome overall, despite the relevant burden (8, 9). Our results support the importance of the caregiver in the device management. The caregiver supported a better patient compliance overall, especially in a later follow-up. Accordingly, the analysis of the final evaluation questionnaire revealed that nearly 20% frequently asked for caregiver's help.

The sample size—which could be appropriated for a pilot study—needs to be improved in a larger prospective study in order to guarantee an adequate representation of the various disease stages and subtypes and to draw more robust conclusions even on app biomarkers. However, this was not a validation study and its primary objective was to evaluate the usability and the compliance of a smartphone app for PD RPM. At the same time, it was possible to associate several quantified motor and cognitive outcomes to available disease severity indexes (disease duration, UPDRS-III total score, H&Y; Supplementary Materials), as also previously reported (10–12). Interestingly some of the motor and cognitive parameters—in particular TUG test data—were not associated to age but specific to the PD condition (Supplementary Table 3). This observation should be replicated in the presence of a control population, which is currently missing. To this regard, the EncephaLog^TM^ TUG test has been already validated against other medical devices in dedicated laboratories and compared to GAITRite pressure walkaway, Vicon 3D cameras and wearables providing a reliable biomarker in both PD and healthy volunteers (12–14).

Mobile-health is on the rise and demonstrates that, in combination with machine learning protocols, it is able to track some of the complex and fluctuating manifestations of PD (15–17).

According to our results, the presence of fluctuations is associated to a more frequent use of the app. In the presented cohort, motor complications were captured only by the UPDRS-IV questionnaire, which provided a dichotomous outcome about the presence/absence of specific motor fluctuations (e.g., sudden offs, unpredictable offs, dyskinesias). Motor fluctuations would have been better tracked by motor diaries. These were not included in the present study, but we acknowledge their essential role in RPM aimed to address motor fluctuations (16, 17).

In conclusion, our study suggests that available technologies can be used for telemedicine, even in a population with limited skills and in a critical situation like a pandemic—which could considerably affect the health of neurological patients directly or indirectly (i.e., worsening of stressors) (18). Some other limitations, such as the brief protocol duration, the absence of controls and the lack of in-person objective measures to compare, warrant further studies to confirm our preliminary findings.

There is still a long-way ahead of us before in-persons visits could be actually seen as “option-B,” since the reliability of new technologies and smartphone apps—released in the most recent years—needs to be proven (19, 20). However, in “emergency conditions”, we found that this combined approach—calls and app—can represent a good compromise to follow-up patient care. New studies are warranted on a larger sample size and for longer periods of time to evaluate the effectiveness of mobile health in patients' management.

## Data Availability Statement

The raw data supporting the conclusions of this article will be made available by the authors, without undue reservation.

## Ethics Statement

The studies involving human participants were reviewed and approved by Comitato etico dell'Università Campus Bio-Medico di Roma. The patients/participants provided their written informed consent to participate in this study.

## Author Contributions

FM wrote the first draft and managed data collections. AM, FP, and MR collected data. FC and VD reviewed the paper for intellectual content. KK, AS-I, and ZY managed data collection and provided IT assistance. MM wrote the first draft, provided statistical analysis ad reviewed the manuscript. All authors contributed to the article and approved the submitted version.

## Conflict of Interest

ZY is co-founder and CEO, AS-I is CSO and KK is CTO of Montfort Brain Monitor LTD a company providing smartphone-based neurological tests. The remaining authors declare that the research was conducted in the absence of any commercial or financial relationships that could be construed as a potential conflict of interest.
