# Supplementary material for: Parkinson's Disease Remote Patient Monitoring During the COVID-19 Lockdown
Source: Front Neurol. 2020 Oct 7;11:567413. doi: 10.3389/fneur.2020.567413 (PMC7575750; doi:10.3389/fneur.2020.567413)
Supplement: Supplementary file 1 [file Data_Sheet_1.docx]

Supplementary Material

**Supplementary methods**

*Specifics of the EncephaLog Home^TM^ application for smartphone.*

*App validation stage*

Montfort has conducted multiple clinical trials in order to validate the correlation between EncephaLog^TM^ output and the scores provided by neurologists monitoring healthy control subjects as well as patients who suffer from PD, multiple sclerosis, normal pressure hydrocephalus, Huntington’s disease and more. Furthermore, the app was validated against other medical devices in dedicated laboratories and compared to GAITRite pressure walkaway, Vicon 3D cameras, wearables, and a digital goniometer.

*Setting up of the monitoring protocol*

Before setting up a remote monitoring protocol, first, the physician who is doing the monitoring installs the EncephaLog Clinic^TM^ (clinician’s app), a process that lasts a couple of minutes. Sending a prescription to EncephaLog Home^TM^ (patient’s app) to the patient requires choosing a predefined test protocol and sending an e-mail or a simple text message to the smartphone. The patient opens the link, the app installs itself as a unique version according to the test protocol and patient’s defined language, and remote monitoring can begin. This process also last a couple of minutes at most. In some cases, a follow up phone call was made to guide the patient through, if there was any difficulty in conducting the test. The physician can alter the test protocol (e.g., choose different tests, change test frequency by a push of a button). If a new test protocol is required (e.g., to address a different neurological condition), this can take up to an hour to set, and then the new protocol is readily available for all future users. Once launched, the app automatically ran the test playlist. The latter was the same for all patients, all along the monitoring program and assembled for the study in order to avoid further variability. Each app is associated to an anonymized code, manually reported by experimenters along with patient demographic information and clinical data on the researcher’s dataset.

*The test playlist*

The test playlist starts with a self-evaluation of the disease status (Parkinson’s status), followed by 8 cognitive tests (Flanker, Memory, Reaction Time, Stroop reading, Stroop word meaning, Stroop Ink color, Trail Making Test A, Trail Making Test B) and 10 task exploring motor functions (Rest Tremor right hand, Rest tremor left hand, Postural tremor right hand, Postural tremor left hand, Tapping test right hand, Tapping test left hand, Balance test on neutral stance, Balance test feet together, two 3 meters Time-Up-and-Go test or TUG). Tests are described in the text below and in supplementary table 1.

*Parkinson’s Status (range 0 – 5)*

The patient was asked to perform an auto-evaluation of his/her disease status in that specific moment of the day ranging from 0 to 5 and to select how long ago he/she took the last PD medication.

*Flanker*

Patients had to indicate the direction of a target arrow presented on the screen. In one condition, a target arrow is flanked by nontarget arrows pointing to the same direction. In another condition, the target arrow is flanked by nontarget arrows pointing randomly. Conditions were randomized during the task. Total accuracy and reaction time were calculated.

*Memory (range 2 – 9)*

The patients visualized on the smartphone a combination of numbers, in progression from 2 to 9, and were asked to tape the combination on the smartphone after each projection.

*Reaction time*

Patients were asked to identify a target picture among a series of images on the screen as quickly as possible. The average reaction time was then calculated.

*Stroop test*

Patients were asked to perform three different tasks. In the reading condition, participants read the names of four colors (red, yellow, green, blue). In the meaning condition, participants named the colors. In the word ink colon condition (interference), participants were asked to name the color of words printed in incongruent colors. Test duration and the average of the corrected answers were calculated for interference condition only.

*Trail Making Test*

Patients were asked to connect a set of numbers (test A) or letters (test B) as quickly as possible. The test was scaled down in a manner that will keep the keys relatively large so they are easy to read and press, while compressing the distance between the keys to fit into the smartphone screen. The time between types can be factored according to the size of the screen (which is known by the app). One can choose up to 20 different dots, connection is made by pressing the dots one after the other.

*Postural tremor test (right and left hand separately)*

Patients were asked to stretch out the arms in front of them while holding the smartphone in their right and left hand for a few seconds (i.e. ~ 10 seconds). Tremor frequency and energy (i.e. the spectral power) were then analyzed.

*Rest tremor test (right and left hand separately)*

Patients were asked to hold the smartphone steady for a few seconds (i.e. ~ 10 seconds) on the palm of their right and left hand, while sitting on a chair with arms leaning on a table. Tremor frequency and energy (i.e. the spectral power) were then analyzed.

*Tapping test (right and left hand separately)*

The tapping test consisted, for each hand, in tapping alternatively with the index finger on two targets located in the right and the left side of the smartphone screen, in landscape mode. A total tapping score was calculated starting from total number of taps and average tap radius (distance between the tap and the target).

*Balance test*

Patients were asked to stand up for a few seconds with the smartphone in landscape mode on their chest (held in position with tape or a belt). Two conditions were explored: i) neutral stance (patients had to stay still on their feet with the stance they felt most comfortable with), ii) feet together (patients were instructed to perform a modified version of Romberg’s test with eyes open).

*3 meters Time Up and Go test (TUG).*

Patients were asked to fix the smartphone on their chest (with a tape or a strap belt) in landscape mode and to perform the 3 meters TUG twice. Putting the smartphone in a strap and putting the strap on to the chest may be challenging to some patients – in that case they could be helped by the caregiver. If the smartphone is located in a strap, the app adjusts for any misalignment in its angle of the strap. Subjects were instructed to perform each test according to the standardized methodology. Instructions were provided on the screen before the test and an audio-guide with countdowns supported the patient during the test.

Results of TUG, tremor, finger tapping, memory span, Stroop interference test, TMT-A and TMT-B are presented in supplementary table 2. The correlation analysis with age, disease duration, UPDRS total score and modified Hoehn and Yahr score are reported in supplementary table 3.

*Visual analog scale (VAS) for patient satisfaction*

The VAS included 6 items, with 5 possible score to each item, ranging from 1 to 5, for evaluation of the experience (not satisfied at all, not satisfied, I don’t know, satisfied, very satisfied), perceived medical supervision (far less supervised, less supervised, no differences, more supervised, much more supervised), difficulties in the use of the app (very difficult, difficult, medium difficulty, easy, very easy), need of support to run the app (every time, most of the times, half times, sometimes, always), burden by app remainder (definitively no, no, I don’t know, yes, definitively yes), wish to continue the remote monitoring (definitively no, no, I don’t know, yes, definitively yes). Patients received the questionnaire by e-mails and were asked to fill the questionnaire and send it back by e-mails or regular mail. Results are reported in supplementary table 4.

**Supplementary table 1.** App tests in their presenting order and possible biomarkers (outcomes).

|  | Test | Outcomes |
| --- | --- | --- |
| Global self-assessment |  |  |
|  | Parkinson’s Status question | Arbitrary Units, range 0 – 5 |
| Cognitive assessment |  |  |
|  | Flanker | Congruent correct (%), incongruent correct (%) |
|  | Memory span | Amount of numers, range 2 – 9 |
|  | Reaction time | Total correct (%), reaction time (s) |
|  | Stroop interference test | Test duration (s), total correct (%), reaction time (s) |
|  | Stroop interference test | Test duration (s), total correct (%), reaction time (s) |
|  | Trail Making Test A | Total correct (%), total time (s) |
|  | Trail Making Test B |  |
| Motor performances |  |  |
|  | Rest tremor right hand | Frequency (Hz), energy (m/s^2^ Hz) |
|  | Rest tremor left hand |  |
|  | Postural tremor right hand |  |
|  | Postural tremor left hand |  |
|  | Tapping test right hand | Number of taps, total score |
|  | Tapping test left hand |  |
|  | Balance test neutral stance | Pitch, yaw, roll (rad./s) |
|  | Balance test feet together | Pitch, yaw, roll (rad./s) |
|  | Time up and go test | Stand-up time (s)  Rotation time (s)  Sit down time (s)  Total time (s)  Walk time (s)  Step in 3 m (n)  Step length (m)  Mediolateral sway (m)  AP step correlation (R^2^)  ML step correlation (R^2^) |

**Supplementary table 2.** Median motor and cognitive outcomes by the app. Data are reported as median (quartiles, QI-QIII).

| **Test** | | **Median (QI-QIII)** |
| --- | --- | --- |
| **3 meters Time up and go test (n=33)** | |  |
|  | Stand-up time (s) | 1.8 (1.5 – 1.97) |
|  | Rotation time (s) | 1.8 (1.4 – 1.97) |
|  | Sit down time (s) | 3.2 (2.6 – 3.92) |
|  | Total time (s) | 15.9 (13.5 – 21.7) |
|  | Walk time (s) | 4.2 (3.4 – 6.2) |
|  | Step in 3 m (n) | 8 (6.5 – 9.7) |
|  | Step length (m) | 0.4 (0.33 – 0.53) |
|  | Mediolateral sway (m) | 0.04 (0.035 – 0.068) |
|  | AP step correlation (R^2^) | 0.33 (0.24 – 0.46) |
|  | ML step correlation (R^2^) | 0.18 (0.34 – 0.5) |
| **Rest tremor (n=27)** | |  |
|  | Rest Tremor Frequency (Hz) | 8.2 (6.1 – 9.3) |
|  | Rest Tremor Energy (m/s^2^ Hz) | 2.7 (1.6 – 4.5) |
| **Postural tremor (n=27)** | | |
|  | Postural Tremor Frequency (Hz) | 7 (5.9 – 8.3) |
|  | Postural Tremor Energy (m/s^2^ Hz) | 6 (4.4 – 7.9) |
| **Finger tapping test (n=37)** | |  |
|  | Total tapping (n) | 18.7 (15 – 25.7) |
|  | Tapping total score | 56.6 (38.6 – 63) |
| **Memory span test (n=39)** | | 6.6 (6 – 6.1) |
| **Stroop interference Test (n=39)** | |  |
|  | Test duration (s) | 32.4 (25.8 – 39.2) |
|  | Total correct (%) | 96 (86.8 – 98.1) |
|  | Reaction time (s) | 1.5 (1.2 – 1.8) |
| **Trail making test A (n=39)** | |  |
|  | Total correct (%) | 99.6 (97.7 – 100) |
|  | Total time (s) | 8.6 (6.7 – 13.8) |
| **Trail making test B (n=39)** | |  |
|  | Total correct (%) | 98 (96.7 – 100) |
|  | Total time (s) | 19.6 (8.4 – 15.6) |

**Supplementary table 3.** Correlation analysis between mean gait parameters, finger tapping, tremor and Stroop interference with age, disease duration, UPDRS-III total score and H&Y of PD patients. Data are reported as correlation coefficient (p-value), *p<0.05. **p<0.01. UPDRS, Unified Parkinson’s Disease Rating Scale; H&Y, modified Hoehn and Yahr score; AP, Anterior-Posterior; ML, Medio-Lateral.

| **Test** | **Age (y)** | **Disease duration (y)** | **UPDRS-III total** | **H&Y** |
| --- | --- | --- | --- | --- |
| **3 meters TUG** |  | | | |
| Stand-up time (s) | -0.025 (0.886) | 0.203 (0.255) | 0.017 (0.924) | 0.296 (0.093) |
| Rotation time (s) | 0.107 (0.552) | 0.153 (0.394) | 0.095 (0.598) | 0.363 (0.037)* |
| Sit down time (s) | 0.013 (0.940) | 0.143 (0.433) | 0.125 (0.495) | 0.243 (0.179) |
| Total time (s) | 0.038 (0.832) | 0.478 (0.005)** | 0.364 (0.040)* | 0.534 (0.000)** |
| Walk time (s) | 0.010 (0.955) | 0.471 (0.006)** | 0.416 (0.017)* | 0.494 (0.004)** |
| Step in 3 m (n) | -0.013 (0.946) | 0.445 (0.010)* | 0.482 (0.005)** | 0.404 (0.021)* |
| Step length (m) | 0.143 (0.425) | -0.423 (0.013)* | -0.392 (0.024)* | -0.247 (0.164) |
| Mediolateral sway (m) | 0.028 (0.878) | 0.325 (0.069) | 0.067 (0.712) | 0.431 (0.013)* |
| AP step correlation (R^2^) | -0.074 (0.700) | -0.432 (0.019)* | -0.325 (0.084) | -0.422 (0.022)* |
| ML step correlation (R^2^) | 0.051 (0.790) | -0.407 (0.028)* | 0.419 (0.023)* | -0.486 (0.007)** |
| **Finger tapping** |  | | | |
| Total taps (n) | -0.137 (0.424) | 0.081 (0.637) | -0.065 (0.703) | 0.062 (0.716) |
| Total score | 0.099 (0.563) | -0.373 (0.024)* | -0.468 (0.003)** | -0.341 (0.041)* |
| **Rest Tremor** |  | | | |
| Frequency (Hz) | -0.357 (0.102) | 0.409 (0.058) | 0.040 (0.999) | 0.057 (0.799) |
| Energy (m/s^2^ Hz) | -0.049 (0.871) | 0.188 (0.387) | 0.177 (0.417) | 0.191 (0.381) |
| **Postural Tremor** |  | | | |
| Frequency (Hz) | -0.366 (0.078) | 0.046 (0.827) | 0.139 (0.516) | 0.204 (0.338) |
| Energy (m/s^2^ Hz) | -0.263 (0.248) | 0.649 (0.001)** | 0.127 (0.582) | 0.156 (0.497) |
| **Stroop interference** |  | | | |
| Test duration (s) | 0.186 (0.256) | -0.001 (0.995) | 0.334 (0.031)* | 0.230 (0.158) |
| Total correct (n) | -0.009 (0.563) | -0.278 (0.086) | -0.362 (0.023)* | -0.207 (0.204) |

**Supplementary table 4.** Results of the final evaluation questionnaire. Few data were missing due to uncomplete or corrupted written feedback by patients.

| **Question** | | **Answer, n, (%)** | |
| --- | --- | --- | --- |
| “Overall, how do you rate the remote-monitoring experience?” (n=44) | |  | |
|  | Utterly disappointing | 0 |  |
|  | Not satisfying | 0 |  |
|  | I don’t know | 7 (15.9) |  |
|  | Satisfying | 16 (36.4) |  |
|  | Very satisfying | 21 (47.7) |  |
| “Did you feel more or less safe in comparison to in-person visit?” (n=43) | |  | |
|  | Much less safe | 1 (2.3) |  |
|  | Less safe | 9 (20.4) |  |
|  | No perceived difference | 12 (27.9) |  |
|  | Safer | 17 (39.5) |  |
|  | Much safer | 4 (9.3) |  |
| “How do you judge the ease of use of the EncephalogHome app ?” (n=43) | |  | |
|  | Much difficult | 0 | |
|  | Difficult | 1 (2.3) | |
|  | Not particularly difficult | 10 (23.2) | |
|  | Easy | 24 (55.8) | |
|  | Very easy | 8 (18.6) | |
| “Did you need help with the use of the app?” (n=43) | |  | |
|  | Every time | 5 (11.6) | |
|  | Many times | 4 (9.3) | |
|  | Half the time | 0 | |
|  | Sometimes | 17 (37.2) | |
|  | Never | 18 (41.8) | |
| “How difficult it was to remember to run the app as requested?” (n=43) | |  | |
|  | Much difficult | 12 (27.9) | |
|  | Difficult | 25 (58.1) | |
|  | Not particularly difficult | 4 (9.3) | |
|  | Easy | 2 (4.6) | |
|  | Very easy | 0 | |
| “If you could, would you continue the remote monitoring program?” (n=50) | |  | |
|  | Absolutely no | 1 (2) | |
|  | Maybe | 3 (6) | |
|  | I don’t know | 5 (10) | |
|  | Yes | 25 (50) | |
|  | Absolutely yes | 16 (32) | |
